# Computed tomography versus magnetic resonance imaging for the perioperative management of acute thoracolumbar intervertebral disc extrusion in non‐ambulatory chondrodystrophic dogs

**DOI:** 10.1111/vsu.70135

**Published:** 2026-07-31

**Authors:** Daniel Low, Andrea Markovic

**Affiliations:** ^1^ Swift Referrals Wetherby UK; ^2^ frank. Pet Surgeons Leeds UK

## Abstract

**Objective:**

To determine whether perioperative management guided by conditional computed tomography‐myelography (C‐CTM) is non‐inferior to magnetic resonance imaging (MRI) regarding functional outcome for acute thoracolumbar intervertebral disc extrusion (IVDE) in non‐ambulatory chondrodystrophic dogs.

**Study design:**

Quasi‐randomized natural experiment utilizing unscheduled MRI downtime as an exogenous instrument.

**Sample population:**

A total of 560 client‐owned dogs (MRI, 518 dogs; C‐CTM, 42 dogs).

**Methods:**

A retrospective cohort study was conducted. The primary outcome was recovery of ambulation, analyzed with intention‐to‐treat (ITT) and per‐protocol (PP) principles with a prespecified non‐inferiority margin of −12%. Secondary outcomes included safety events (postoperative neurologic deterioration, progressive myelomalacia, major complications) and time‐adjusted treatment costs.

**Results:**

C‐CTM was non‐inferior to MRI for recovery of ambulation in both ITT (risk difference + 6.5%, 95% CI: −2.7%–15.7%, one‐sided *p* < .001) and PP analyses (risk difference + 2.5%, 95% CI: −7.3%–12.2%, one‐sided *p* < .001). There was no difference in rates of postoperative neurologic deterioration, progressive myelomalacia, or major complications. Total treatment costs and imaging costs were significantly lower for C‐CTM (*p* < .01). There was no difference in non‐imaging costs between groups.

**Conclusion:**

In non‐ambulatory chondrodystrophic dogs with acute thoracolumbar IVDE, C‐CTM is non‐inferior to MRI for recovery of ambulation with a similar safety profile, at significantly lower cost.

**Clinical significance:**

In dogs with a high pretest probability of acute thoracolumbar IVDE, routine use of MRI may not be justified given the comparable clinical outcomes and lower treatment costs achieved with C‐CTM.

## 1 INTRODUCTION

Thoracolumbar intervertebral disc extrusion (IVDE) is a leading cause of acute paraparesis or paraplegia in dogs.^1^, ^2^ Cross‐sectional imaging techniques such as non‐contrast computed tomography (CT), CT‐myelography, and magnetic resonance imaging (MRI), are all reasonable for diagnosis and treatment planning.^3^ Non‐contrast CT is most sensitive in chondrodystrophic dogs with mineralized extruded disc material, with the benefit of shorter scan times and wider availability.^4^ CT‐myelography may be required to demonstrate extradural compression if non‐contrast CT is non‐diagnostic, with this procedure having been associated with post‐myelography adverse events.^5^ MRI is regarded as the reference standard for imaging canine intervertebral disc disease,^6^ but is disadvantaged by prolonged scan times and is less accessible to pet owners because of the resultant increase in costs.^3^

Parry and others^7^ performed a retrospective comparison of myelography and MRI in dogs with acute thoracolumbar myelopathy and did not observe any clinical benefit with the use of MRI. A prospective comparison of CT and MRI for planning of decompressive hemilaminectomy has been performed, although evaluation of clinical outcomes was not within the scope of the study.^8^ MRI may be useful in guiding treatment decisions regarding durotomy,^3^ though the role of durotomy remains far from definitive.^9^ Beyond diagnosis and treatment planning, MRI has been promoted for its prognostic potential especially for deep‐pain‐negative dogs with severe acute spinal cord injury, however evidence linking imaging biomarkers to clinically meaningful outcomes remains inconsistent. Several studies report that the presence of T2‐weighted intramedullary hyperintensity is associated with postoperative functional outcome^10^, ^11^, ^12^ but other studies have not replicated these findings.^13^, ^14^, ^15^ Certain MRI biomarkers have been associated with risk of progressive myelomalacia (PMM) but existing studies are limited by small case numbers and the inconsistent timing of image acquisition.^16^, ^17^, ^18^ This makes it challenging to establish a relationship between MRI biomarkers and risk of PMM, as the natural progression of acute severe spinal cord injury is also poorly understood.^19^ The choice of imaging modality has no direct influence on biological recovery but may affect outcomes indirectly through its impact on surgical planning and clinical decision‐making.

In dogs with thoracolumbar myelopathy, the use of CT as a first‐line imaging modality is appropriate^20^ but in the last decade, the use of MRI to manage acute thoracolumbar IVDE is becoming increasingly widespread in specialty practice, with a shift to almost exclusive use of MRI by neurologists and increasing routine use of MRI by surgeons.^1^, ^21^ The reasons for this shift are likely multifactorial with the perception that MRI the additional information offered by MRI improves outcomes being a contributing factor. Despite this shift, there is a remarkable lack of evidence directly comparing clinical outcomes in dogs imaged with CT or MRI, with serious implications regarding access to care if MRI is prioritized as a first‐line imaging modality.^21^

This study aimed to perform a non‐inferiority comparison of CT versus MRI for the perioperative management of acute thoracolumbar IVDE in non‐ambulatory chondrodystrophic dogs. The primary null hypothesis was that perioperative management guided by CT would result in a lower rate of recovery of ambulation than MRI‐guided management by more than the pre‐specified non‐inferiority margin. A secondary analysis of safety compared the risk of postoperative neurologic deterioration, development of PMM, and risk of postoperative complications. Finally, a comparative analysis of imaging and total treatment costs was performed.

## 2 METHODS

### 2.1 Study design

This study was reported in accordance with the “Transparent Reporting of Evaluations with Nonrandomized Designs” statement (Data S1).^22^ All data in this study was obtained retrospectively from a single referral hospital in the United Kingdom with written client consent granting permission for the anonymized use of clinical data for research purposes. A natural experiment was conducted whereby unscheduled MRI scanner downtime was used as an exogenous instrument allocating dogs to conditional CT‐myelography (C‐CTM) in a quasi‐randomized fashion. At the authors' institution, MRI is the default imaging modality for dogs with acute thoracolumbar myelopathy. During periods of unscheduled MRI scanner downtime, C‐CTM was performed instead.^23^

The flow of dogs through screening, exclusion, and analysis is detailed in Figure 1. Initial screening criteria aimed to identify chondrodystrophic dogs with a clinical^24^ or cross‐sectional imaging^25^ diagnosis of acute thoracolumbar IVDE, defined as clinical signs <10 days in duration.^26^ Chondrodystrophic dogs were defined according to previous studies.^27^, ^28^ From the screened population, dogs were excluded if they were ambulatory, managed conservatively without imaging, or euthanized without imaging. All MRI studies were acquired with a Siemens Aera 1.5‐Tesla (Munich, Germany). A minimum of T2‐weighted sagittal and T2‐weighted transverse sequences with slice thickness of 2.5 mm and field of view of 2.75 mm were obtained, with additional sequences at the discretion of the attending surgeon. All CT studies were performed using a General Electric Optima CT660 128‐slice (Boston, Massachusetts) with slice thickness of 1.25 mm, and reconstructed with standard bone and soft tissue algorithms. When indicated, myelography using 0.2–0.3 mL/kg iohexol (Omnipaque 240 mg iodine/ml; GE Healthcare, Chicago, Illinois) was performed in the same anesthetic episode, via cisternal or lumbar puncture. All imaging studies were performed under general anesthesia. Qualitative validity of quasi‐randomization was established through review of MRI service logs and corroboration with clinical records, to verify that CT and CT‐myelography were performed due to MRI scanner downtime and not for other reasons. All remaining dogs constituted the intention‐to‐treat (ITT) population. The ITT population included dogs that were conservatively managed after imaging, that were euthanized after imaging, and that underwent decompressive surgery. Dogs undergoing decompressive surgery which did not have outcome data available were analyzed within the ITT group only. Dogs within the ITT group either underwent MRI by default (ITT‐MRI) or were quasi‐randomized to C‐CTM during MRI downtime (ITT‐CT). The per‐protocol (PP) population was a subset of the ITT population, restricted to dogs that underwent decompressive hemilaminectomy and which had outcome data available. Dogs within the PP group either underwent MRI by default (PP‐MRI) or were quasi‐randomized to C‐CTM during MRI downtime (PP‐CT). Dogs experiencing multiple discrete episodes of IVDE were included more than once if full functional recovery occurred between episodes. Each episode was treated as an independent clinical event. In dogs undergoing surgical management of acute thoracolumbar IVDE, standard decompressive hemilaminectomy was performed, with adjunctive procedures such as fenestration or durotomy performed at the attending surgeon's discretion. Dogs were hospitalized postoperatively for multimodal analgesia with opioids, non‐steroidal anti‐inflammatory drugs, paracetamol, and gabapentin, and for bladder management. Dogs were discharged with multimodal oral analgesia with non‐steroidal anti‐inflammatory drugs, paracetamol, and gabapentin, and with instructions for 6 weeks of cage rest.

**FIGURE 1:**
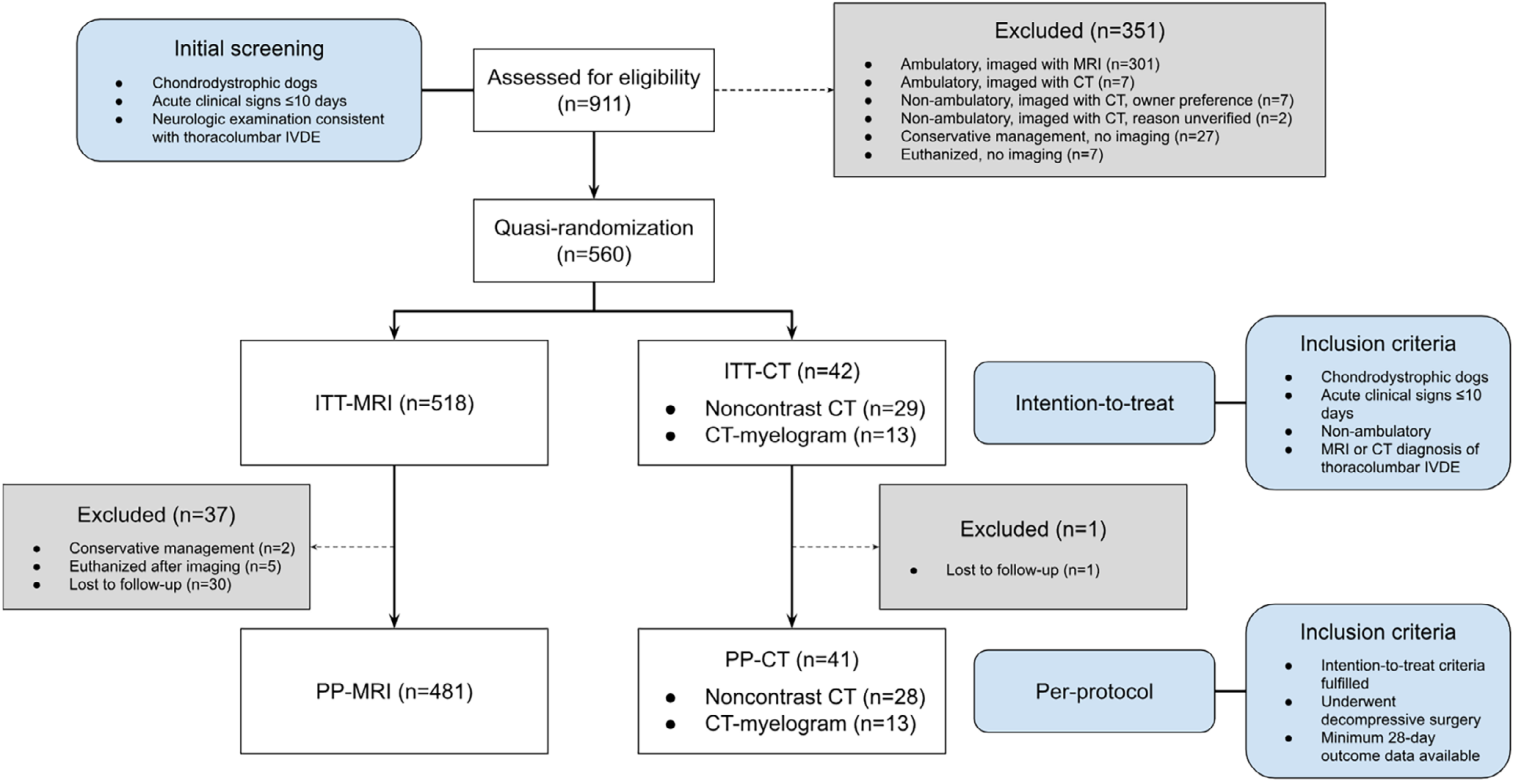
Flow diagram of initial screening, quasi‐randomization, progression through the intention‐to‐treat and per‐protocol analyses, and exclusions.

### 2.2 Data extraction and outcome definition

Retrospective data extraction was performed via manual search of electronic health records and practice management invoice records from 2019 to 2025. Neurologic grade at presentation was assessed according to the ascending modified Frankel score; ambulatory status was based on functional clinical assessment, as determined by the managing clinician.^19^, ^29^ Preoperative clinical data collected included imaging modality, neurologic grade, attending surgeon, surgeon type (diplomate, resident under direct supervision, or non‐boarded clinician), signalment, bodyweight, time from onset of clinical signs to surgery, time from referral consultation to surgery, craniocaudal extent of hemilaminectomy (recorded as the number of articular facets removed), use of fenestration and number of intervertebral discs fenestrated, and the use and extent of durotomy. Postoperative clinical data collected included myelography‐related adverse events, postoperative neurologic grade to assess for postoperative neurologic deterioration, postoperative complications, time to follow‐up, and method of follow‐up. Specific complications recorded included the need for revision decompressive surgery, development of PMM, and death or euthanasia unrelated to PMM. Revision decompressive surgery was defined as re‐operation within 28 days as a result of de novo or residual extradural compression at the same site. The invoiced costs of diagnostic imaging and the total cost of treatment of the IVDE episode were collected from the practice management system.

Recovery of ambulation was defined as the primary outcome, defined as functional recovery. Outcome data was obtained at a minimum of 28 days via follow‐up neurologic examination or via remote video assessment. Secondary safety outcomes were included to evaluate whether differences in imaging‐derived information may be associated with differences in downstream clinical decision‐making, including surgical planning, treatment selection, and the use of adjunctive procedures. Three categories of secondary safety outcomes were analyzed, (1) postoperative neurologic deterioration, (2) development of PMM, and (3) postoperative complications. Postoperative neurologic deterioration was defined as an increase in one or more neurologic grades on the modified Frankel scale. Progressive myelomalacia was diagnosed with compatible clinical signs.^17^ Postoperative complications were analyzed as a composite of the need for revision decompressive surgery, development of PMM, and death or euthanasia unrelated to PMM. Postoperative complications were collected up until the point of follow‐up assessment. Invoiced costs from the practice management system were compared between groups. Total treatment costs, total diagnostic imaging costs, and total non‐imaging treatment costs in pounds sterling were separately analyzed.

### 2.3 Statistical analysis

Based on a power calculation with expected proportions of 0.90, an allocation ratio of 10, a one‐sided alpha of 2.5%, a non‐inferiority margin of −12%, and study power of 80% for the primary outcome, a minimum group size of 41 dogs was required. The time from onset of signs to surgery and time from presentation to surgery were compared between groups using the Wilcoxon rank‐sum test. Quasi‐randomization was quantitatively validated by comparing baseline covariates between C‐CTM and MRI groups by calculating the standardized mean difference (SMD). Consistent with established convention, an SMD of < 0.1 was taken as an indication of negligible difference between groups.^30^ Robustness of primary analyses to potential unmeasured confounding was additionally assessed by the E‐value. The primary analysis tested non‐inferiority of C‐CTM to MRI in both ITT and PP groups, for recovery of ambulation using a −12% margin. Primary testing used unadjusted risk differences with supportive multivariable logistic regression adjusting for neurologic grade, surgeon type, and patient age. In ITT analysis only, robustness of findings was assessed through sensitivity analyses using (1) complete‐case analysis excluding dogs lost to follow‐up, and (2) additional adjustment for covariates with SMDs > 0.1. In ITT analysis, dogs without outcome data were handled conservatively and considered as failures (did not recover ambulation) without complications (no postoperative neurologic deterioration, no PMM, and no postoperative complications). Secondary safety outcomes, including postoperative neurologic deterioration, development of PMM, and postoperative complications, were compared between groups with Fisher's exact test. Results of ITT and PP analysis were expressed as unadjusted risk differences and adjusted ORs, with associated 95% CIs. Subgroup analysis was performed on deep‐pain‐positive dogs as described above, to assess clinical outcomes in the deep‐pain‐positive subgroup where postoperative recovery rates are similar. To assess the impact of imaging modality on surgical planning, hemilaminectomy window size, fenestration variables, and durotomy variables were compared between groups using Wilcoxon rank‐sum tests for continuous variables and Fisher's exact tests for categorical variables. To assess the association between the use of same‐site fenestration and risk of revision decompressive surgery in the 28‐day follow‐up period, Fisher's exact test was used. Further sensitivity analysis for independence of observations was performed excluding further episodes from dogs contributing more than one episode.

Economic analysis was pre‐specified and contingent on non‐inferiority of the primary outcome. In the event that non‐inferiority was established for recovery of ambulation, a cost‐minimization analysis was planned. In the event that non‐inferiority was not established, a cost–benefit analysis was planned. To account for non‐clinical confounders such as inflation and fluctuating hospital pricing over the six‐year study period, a time‐adjusted cost analysis was performed. For each quarter, the quarter‐specific median cost of treatment was determined. The median value of the quarter‐specific median costs was defined as the reference and an adjustment factor applied, effectively scaling costs to the economic conditions of the reference period. Time‐adjusted total treatment costs, imaging costs, and non‐imaging costs were compared between groups using Wilcoxon rank‐sum tests.

Data normality was assessed with the Shapiro–Wilk test. Nonparametric data are expressed as median and interquartile range (IQR) and parametric data expressed as mean and SD. Statistical significance was defined as a *p*‐value of less than .05. All statistical analyses were performed with dplyr 1.1.4, tidyr 1.3.1, tableone 0.13.2, broom 1.0.10, tidyverse 2.0.0, and lubridate 1.9.4 in R version 4.5.2.^31^, ^32^, ^33^, ^34^, ^35^, ^36^

## 3 RESULTS

### 3.1 Study enrolment

Enrolment and exclusions are shown in Figure 1. Initial screening identified consecutive records of 911 chondrodystrophic dogs presenting with clinical signs and a neurologic examination consistent with acute thoracolumbar IVDE. Dogs which were ambulatory (*n* = 308), imaged with CT but did not fulfill quasi‐randomization criteria (*n* = 9), managed conservatively without imaging (*n* = 27), or euthanized without imaging (*n* = 7) were excluded. Of the nine dogs imaged with CT not fulfilling quasi‐randomization criteria, owner preference was the recorded reason in seven dogs and the reason could not be verified in the other two dogs. Of the 27 dogs managed conservatively without imaging, 13 were neurologic grade 1, nine were neurologic grade 2, four were neurologic grade 3, and one was neurologic grade 4. Of the seven dogs euthanized without imaging, one was neurologic grade 2, one was neurologic grade 3, and five were neurologic grade 5.

After exclusions from initial screening, 560 non‐ambulatory chondrodystrophic dogs underwent cross‐sectional imaging and constituted the ITT group, with 518 dogs in ITT‐MRI and 42 dogs in ITT‐CT (non‐contrast CT, *n* = 29; CT‐myelogram, *n* = 13). Of these observations, 23 dogs (4.4% of observations) within ITT‐MRI had more than one episode of IVDE (two episodes, *n* = 21; three episodes, *n* = 2). Qualitative validity of quasi‐randomization in ITT‐CT was confirmed through review of MRI service logs and corroboration with clinical records. The attending surgeon was an ECVS diplomate in 163/560 (29.1%) dogs, a resident under direct supervision in 111/560 (19.8%) dogs, and a non‐boarded clinician in 286/560 (51.1%) dogs. Two dogs were managed conservatively after MRI; these dogs were neurologic grade 3 and neurologic grade 4 respectively. Five dogs were euthanized after MRI; one dog was neurologic grade 3 and four dogs were neurologic grade 5. In total, 511 dogs in ITT‐MRI underwent decompressive surgery; outcome data was unavailable for 30 of these dogs (neurologic grade 3, *n* = 11; neurologic grade 4, *n* = 14; neurologic grade 5, *n* = 5). No dogs in ITT‐CT underwent conservative management or were euthanized after CT. All 42 dogs in ITT‐CT underwent decompressive surgery; outcome data was unavailable for one dog (neurologic grade 3). After further exclusions, there were 481 dogs in PP‐MRI and 41 dogs in PP‐CT (non‐contrast CT, *n* = 28; CT‐myelogram, *n* = 13).

### 3.2 Clinical data and postoperative outcomes

Baseline characteristics of the ITT and PP groups are presented in Table 1. Baseline covariate SMDs were < 0.1 for all covariate comparisons except for sex and time from onset of clinical signs to surgery. Surgical treatments, representing perioperative decision‐making, were not significantly different between PP‐MRI and PP‐CT (Table 2). There were no post‐myelographic adverse events recorded in the 13 dogs undergoing CT‐myelography. Postoperative neurologic deterioration was recorded in 61/481 (12.7%) dogs in PP‐MRI and 4/41 (9.8%) dogs in PP‐CT. Of dogs with postoperative neurologic deterioration, 24/61 (39.3%) dogs in PP‐MRI and 3/4 (75.0%) dogs in PP‐CT lost deep pain sensation.

**TABLE 1:** Baseline characteristics by imaging modality in ITT and PP groups, with ITT covariate SMDs.

	ITT‐MRI (*n* = 518)	ITT‐CT (*n* = 42)	SMD	PP‐MRI (*n* = 481)	PP‐CT (*n* = 41)
Age (years)	4.8 (3.8–6.0)	4.8 (3.8–6.1)	0.015	4.8 (3.8–6.0)	4.8 (3.7–6.2)
Bodyweight (kg)	8.5 (6.2–12.1)	7.5 (5.8–12.5)	0.041	8.5 (6.2–12.2)	7.5 (5.7–12.8)
Sex	310 male (187 neutered)	25 male (12 neutered)	**0.117**	288 male (174 neutered)	25 male (12 neutered)
	208 female (137 neutered)	17 female (10 neutered)		193 female (130 neutered)	16 female (9 neutered)
Breed	Dachshund (*n* = 244)	Dachshund (*n* = 17)	0.098	Dachshund (*n* = 224)	Dachshund (*n* = 16)
	Crossbreed (*n* = 96)	Crossbreed (*n* = 9)		Crossbreed (*n* = 91)	Crossbreed (*n* = 9)
	French Bulldog (*n* = 92)	French Bulldog (*n* = 5)		French Bulldog (*n* = 85)	French Bulldog (*n* = 5)
	English Cocker Spaniel (*n* = 32)	English Cocker Spaniel (*n* = 2)		English Cocker Spaniel (*n* = 29)	English Cocker Spaniel (*n* = 2)
	Others (*n* = 54)	Others (*n* = 9)		Others (*n* = 52)	Others (*n* = 9)
Neurologic grade	Grade 3 (*n* = 273)	Grade 3 (*n* = 21)	0.054	Grade 3 (*n* = 260)	Grade 3 (*n* = 20)
	Grade 4 (*n* = 171)	Grade 4 (*n* = 16)		Grade 4 (*n* = 156)	Grade 4 (*n* = 16)
	Grade 5 (*n* = 74)	Grade 5 (*n* = 5)		Grade 5 (*n* = 65)	Grade 5 (*n* = 5)
Time from onset of signs to surgery (days)	2.0 (2.0–3.0)	2.0 (1.0–4.0)	**0.106**	2.0 (2.0–3.0)	2.0 (1.0–4.5)
Time from referral presentation to surgery (days)	0.0 (0.0–1.0)	0.0 (0.0–1.0)	0.027	0.0 (0.0–1.0)	0.0 (0.0–1.0)
Surgeon type	ECVS diplomate (*n* = 150)	ECVS diplomate (*n* = 13)	0.013	ECVS diplomate (*n* = 139)	ECVS diplomate (*n* = 12)
	Resident (*n* = 102)	Resident (*n* = 9)		Resident (*n* = 95)	Resident (*n* = 9)
	Non‐boarded clinician (*n* = 266)	Non‐boarded clinician (*n* = 20)		Non‐boarded clinician (*n* = 247)	Non‐boarded clinician (*n* = 20)

*Note*: Covariates with SMD > 0.1 highlighted in bold. Continuous data are presented as median (IQR). Categorical data reported as counts.

Abbreviations: CT, computed tomography; ECVS, European College of Veterinary Surgeons; ITT, intention‐to‐treat; IQR, interquartile range; MRI, magnetic resonance imaging; PP, per‐protocol; SMD, standardized mean difference.

**TABLE 2:** Summary of surgical treatments performed in PP‐MRI and PP‐CT groups.

	PP‐MRI (*n* = 481)	PP‐CT (*n* = 41)	*p*‐value
Hemilaminectomy spaces			
Number of articular facets removed	1.0 (1.0–2.0)	1.0 (1.0–1.5)	.551
Single‐level decompression	344 (71.5%)	31 (75.6%)	.718
Multi‐level decompression	137 (28.5%)	10 (24.4%)	
Adjunctive procedures			
Fenestration performed	157 (32.6%)	10 (24.4%)	.745
Number of discs fenestrated	0 discs: 324 (67.4%)	0 discs: 31 (75.6%)	.287
	1 disc: 128 (26.6%)	1 disc: 8 (19.5%)	
	2 discs: 23 (4.8%)	2 discs: 2 (4.9%)	
	≥3 discs: 6 (1.2%)	≥3 discs: 0 (0%)	
Durotomy performed	6 (1.2%)	1 (2.4%)	.438
Vertebral body lengths of durotomy	None: 475 (98.8%)	None: 40 (97.6%)	.530
	1 body: 4 (0.8%)	1 body: 1 (2.4%)	
	2 bodies: 1 (0.2%)	2 bodies: 0 (0%)	
	≥3 bodies: 1 (0.2%)	≥3 bodies: 0 (0%)	

*Note*: Continuous data are presented as median (IQR). Categorical data reported as counts (percentages).

Abbreviations: CT, computed tomography; IQR, interquartile range; MRI, magnetic resonance imaging; PP, per‐protocol.

Follow‐up was obtained at a median of 46 days (IQR: 39–64 days) in PP‐MRI and 43 days (IQR: 36–60.5 days) in PP‐CT. Follow‐up was via neurologic examination in 350/481 (72.8%) dogs and remotely in 131/481 (27.2%) dogs in PP‐MRI. Follow‐up was via neurologic examination in 24/41 (58.5%) dogs and remotely in 17/41 (41.5%) dogs in PP‐CT. Rates of recovery of ambulation across neurologic grades and between the ITT and PP groups are presented in Table 3. The rate of PMM was 15/481 (3.1%) dogs in PP‐MRI and 0/41 (0.0%) dogs in PP‐CT. There were 35/481 (7.3%) postoperative complications in PP‐MRI; these included revision decompressive surgery in 20 dogs, PMM in 15 dogs, and death or euthanasia unrelated to PMM in seven dogs, with some dogs having more than one complication. There were 2/41 (4.9%) postoperative complications in PP‐CT; both were revision decompressive surgeries, with no dogs developing PMM or experiencing death or euthanasia in the follow‐up period. Performing same‐site fenestration was not associated with the risk of revision decompressive surgery in the 28‐day follow‐up period (OR 1.78, 95% CI: 0.638–4.83, *p* = .225).

**TABLE 3:** Rates of recovery of ambulation in ITT and PP groups, reported as counts (percentages).

	ITT‐MRI (*n* = 518)	ITT‐CT (*n* = 42)	PP‐MRI (*n* = 481)	PP‐CT (*n* = 41)
Neurologic grade 3	252/273 (92.3%)	20/21 (95.2%)	252/260 (96.9%)	20/20 (100.0%)
Neurologic grade 4	147/171 (86.0%)	15/16 (93.8%)	147/156 (94.2%)	15/16 (93.8%)
Neurologic grade 5	35/74 (47.3%)	3/5 (60.0%)	35/65 (53.8%)	3/5 (60.0%)

Abbreviations: CT, computed tomography; ITT, intention‐to‐treat; MRI, magnetic resonance imaging; PP, per‐protocol.

### 3.3 ITT and PP analyses

Analyses and effect sizes are presented in Table 4. In ITT analysis, C‐CTM was non‐inferior to MRI for recovery of ambulation (unadjusted risk difference + 6.5%, 95% CI: −2.7%–15.7%, one‐sided *p* < .001), exceeding the −12% pre‐specified non‐inferiority margin. The non‐inferiority finding was unchanged after multivariable adjustment (adjusted OR 1.41, 95% CI: 0.41–6.66; *p* = .617). This finding was robust to sensitivity analyses adjusting for sex and time‐to‐surgery covariates (adjusted OR 1.57, 95% CI: 0.45–7.62) and complete‐case multivariable analysis (adjusted OR 1.41, 95% CI: 0.41–6.66). Safety profiles between ITT‐MRI and ITT‐CT were comparable, with no significant differences in rates of postoperative neurologic deterioration, PMM, or postoperative complications.

**TABLE 4:** Summary of postoperative outcomes and safety data in ITT and PP groups, with unadjusted and adjusted effect sizes and associated *p*‐values.

	ITT‐MRI (*n* = 518)	ITT‐CT (*n* = 42)	ITT effect size (95% CI)		PP‐MRI (*n* = 481)	PP‐CT (*n* = 41)	PP effect size (95% CI)	
Primary outcome								
Recovery of ambulation	434/518 (83.8%)	38/42 (90.5%)	Risk difference + 6.5% (95% CI: −2.7%–15.7%)	**One‐sided *p* < .001**	434/481 (90.2%)	38/41 (92.7%)	Risk difference + 2.5% (95% CI: −7.3%–12.2%)	**One‐sided *p* < .001**
			Adjusted odds ratio 1.41 (95% CI: 0.41–6.66)	*p* = .617			Adjusted odds ratio 1.33 (95% CI: 0.385–6.32)	*p* = .681
Secondary safety outcomes								
All postoperative neurologic deterioration	61/518 (11.8%)	4/42 (9.5%)	Risk difference − 2.3% (95% CI: −12.8%–8.3%)	*p* = .851	61/481 (12.7%)	4/41 (9.8%)	Risk difference − 2.9% (95% CI: −12.5%–6.6%)	*p* = .805
Severe neurologic deterioration (loss of deep pain sensation)	24/518 (4.6%)	3/42 (7.1%)	Risk difference 2.5% (95% CI: −6.8%–11.8%)	*p* = .722	24/481 (5.0%)	3/41 (7.3%)	Risk difference 2.3% (95% CI: −5.9%– 10.5%)	*p* = .461
PMM	15/518 (2.9%)	0/42 (0.0%)	Risk difference − 2.9% (95% CI: −5.6%–0.2%)	*p* = .535	15/481 (3.1%)	0/41 (0.0%)	Risk difference − 3.1% (95% CI: −4.7% to −1.6%)	*p* = .621
Postoperative complications	35/518 (6.8%)	2/42 (4.8%)	Risk difference − 2% (95% CI: −10.1%–6.1%)	*p* = .859	35/481 (7.3%)	2/41 (4.9%)	Risk difference − 2.4% (95% CI: −9.4%–4.6%)	*p* = .758

*Note*: Unadjusted risk differences (95% CI) and adjusted OR (95% CI) expressed for CT group relative to MRI group. Categorical data reported as numbers (percentage). Statistical significance was defined as a *p*‐value of less than .05 (in bold).

Abbreviations: CT, computed tomography; ITT, intention‐to‐treat; MRI, magnetic resonance imaging; PMM, progressive myelomalacia; PP, per‐protocol.

In PP analysis, C‐CTM was non‐inferior to MRI for recovery of ambulation (unadjusted risk difference + 2.5%, 95% CI: −7.3%–12.2%, one‐sided *p* < .001), exceeding the −12% pre‐specified non‐inferiority margin. The non‐inferiority finding was unchanged after multivariable adjustment (adjusted OR 1.33, 95% CI: 0.385–6.32; *p* = .681). Safety profiles between PP‐MRI and PP‐CT were comparable, with no significant differences in rates of postoperative neurologic deterioration, PMM, or postoperative complications.

In PP subgroup analysis of non‐ambulatory deep‐pain‐positive dogs (*n* = 452), C‐CTM was non‐inferior to MRI for recovery of ambulation (unadjusted risk difference + 1.3%, 95% CI: −4.4%–7.0%, one‐sided *p* < .001). Safety profiles between the PP subgroups of non‐ambulatory deep‐pain‐positive dogs were comparable, with no significant differences in rates of postoperative neurologic deterioration, PMM, or postoperative complications.

E‐value analysis for the primary outcome (adjusted OR = 1.41) yielded an E‐value of 1.66, indicating that unmeasured confounders would need risk ratios of at least 1.66 with both imaging exposure and outcome to fully explain away the observed treatment effect. Sensitivity analysis excluding second or further episodes showed no change to ITT and PP results.

### 3.4 Time‐adjusted economic analysis

Given the non‐inferiority established in both ITT and PP analyses, cost‐minimization analysis was performed (Table 5). C‐CTM had significantly lower total treatment costs compared to MRI in both ITT (*p* = .006) and PP (*p* = .005) comparisons, and lower imaging costs in both ITT (*p* < .001) and PP (*p* < .001) comparisons. Non‐imaging costs were not different between ITT (*p* = .124) and PP (*p* = .133) comparisons.

**TABLE 5:** Summary of time‐adjusted imaging costs, non‐imaging costs, and total treatment costs between ITT and PP groups, in pounds sterling.

	ITT‐MRI (*n* = 518)	ITT‐CT (*n* = 42)	*p*‐value	PP‐MRI (*n* = 481)	PP‐CT (*n* = 41)	*p*‐value
Imaging costs	2117.59 (1741.5–2117.59)	1085.40 (1026–1259.29)	***p* < .001**	1960.74 (1772.69–2148.79)	1005 (967–1043)	***p* < .001**
Non‐imaging costs	6241.67 (5854.34–6508.51)	6568.44 (4899–7515.01)	*p* = .124	6253.54 (5926.45–6580.63)	6568.44 (5260.44–7876.44)	*p* = .133
Total treatment costs	8250 (7572.76–8500)	8176.61 (6000–8500)	***p* = .006**	8250 (7803.13–8696.87)	7518.44 (6268.44–8768.44)	***p* = .005**

*Note*: Continuous data are presented as median (IQR). Statistical significance was defined as a *p*‐value of less than .05 (in bold).

Abbreviations: CT, computed tomography; IQR, interquartile range; ITT, intention‐to‐treat; MRI, magnetic resonance imaging; PP, per‐protocol.

## 4 DISCUSSION

This quasi‐randomized natural experiment demonstrated that C‐CTM was non‐inferior to MRI for perioperative management of non‐ambulatory chondrodystrophic dogs with acute thoracolumbar IVDE, with regards to the primary outcome of recovery of ambulation, and the primary null hypothesis was rejected. There was no difference in safety profile between C‐CTM and MRI. Total treatment costs and imaging costs were significantly lower with C‐CTM.

This study addresses a critical evidence gap in veterinary neurosurgery. Despite the increasing adoption of MRI over the past decade, there is no evidence showing that the use of MRI leads to better clinical outcomes.^21^ The only other comparative outcomes study in canine spinal disease compared myelography to MRI in dogs with acute thoracolumbar myelopathy (over 80% of which were due to disc disease),^7^ with limited relevance to current practice because of the declining use of myelography. This study replicates their findings and once again shows that the use of MRI reliably escalates costs without an associated improvement in patient‐centered outcomes. This study compares MRI to C‐CTM and provides contemporary evidence that clinical outcomes remain equivalent in this specific, surgically relevant population of non‐ambulatory chondrodystrophic dogs. In light of ongoing concerns regarding the cost of veterinary care,^37^, ^38^, ^39^ prioritizing MRI in non‐ambulatory chondrodystrophic dogs as an aggregate group may not be justified and may represent an unnecessary barrier to accessing care.

A retrospective observational study comparing MRI versus C‐CTM would be inevitably confounded by clinician selection bias, making it difficult to separate the effect of imaging from other factors influencing choice of imaging modality.^21^ To address this limitation, this study used a natural experiment design with MRI downtime as an exogenous instrument allocating dogs to C‐CTM. Natural experiments are a hybrid of observational studies and randomized trials, filling evidence gaps where randomization is ethically or logistically challenging.^40^ This study's results relied on the assumption that MRI downtime is a truly exogenous instrument allocating dogs to C‐CTM. Scanner downtime has not been previously used as a quasi‐randomization tool in human or veterinary medicine, nor has a natural experiment been conducted in small animal surgery. Parry and others^7^ utilized an exogenous factor, the variable availability of mobile MRI, in their retrospective comparison of MRI versus myelography, although their study was not explicitly designed as a natural experiment. In this study, we verified instrument validity qualitatively through corroboration of MRI service logs and clinical records, and quantitatively through assessment of baseline covariate balance. Standardized mean differences between ITT groups showed good to excellent covariate balance, and there was no evidence that the ITT‐CT group was systematically different to the ITT‐MRI group. Where minor imbalances were found in two covariates, multivariable regression in sensitivity analyses showed that these imbalances did not affect results. The robustness of the primary analysis is further supported by sensitivity analysis for unmeasured confounding. The E‐value of 1.66 showed that to negate the observed treatment effect, an unmeasured confounder would need to be strongly associated with both allocation to C‐CTM and recovery of ambulation by risk ratios of at least 1.66 each. In conjunction with the observed balance in measured baseline covariates, it is unlikely that confounding of this magnitude exists. Nonetheless, residual unmeasured endogeneity at the referral hospital level remains possible. During periods of MRI downtime, pre‐hospital factors^26^ leading to case redirection may have been present. For example, referring veterinarians aware of the out‐of‐service MRI scanner may have selectively redirected certain cases to other hospitals, or alternatively, clinicians in the studied referral hospital may have selectively triaged certain cases away during such periods of MRI downtime. These triage patterns cannot be captured retrospectively and would have affected the assumption of quasi‐randomization.

In this study, data analysis followed principles used in randomized trials to maximize the validity of causal inference from the natural experiment design.^41^ The original quasi‐randomized groups are preserved in ITT analysis regardless of protocol adherence to estimate the “real‐world” effect of being allocated to MRI versus C‐CTM, including the effect of all downstream clinical decisions, which may be surgical management, conservative management, or euthanasia. The PP analysis complements this by assessing the effect of imaging on surgical planning and clinical outcomes in protocol‐adherent dogs. The consistent findings of both the ITT and PP analyses robustly support this study's main conclusion that C‐CTM is non‐inferior to MRI where recovery of ambulation is concerned. A noninferiority study was performed instead of superiority testing, as a failure to demonstrate superiority of MRI over C‐CTM could reflect insufficient statistical power rather than true equivalence. In contrast, noninferiority testing allows an affirmative conclusion that C‐CTM performs within a prespecified margin of MRI, thereby supporting its use as an alternative imaging strategy. This approach aligns with contemporary comparative effectiveness research, where the goal is to identify interventions that achieve similar outcomes with lower resource utilization. Importantly, this study was only powered for the primary outcome of recovery of ambulation and not powered for the rarer secondary safety outcomes. While the similar point estimates for safety outcomes across both ITT and PP analyses are reassuring, this study lacks statistical power to definitively rule out small differences in rare complications. This study evaluated non‐ambulatory dogs as an aggregate group and lacked statistical power to evaluate the subgroup of neurologic grade 5 dogs. Subgroup analysis showed that in dogs with neurologic grades 3 and 4, the use of MRI or C‐CTM did not lead to different outcomes or a different safety profile. In non‐ambulatory deep‐pain‐positive dogs, recovery rates after surgical decompression are already known to be high regardless of imaging modality.^42^ This creates a statistical “ceiling effect,” making it methodologically challenging to design a trial with sufficient power to demonstrate the superiority of MRI, as any incremental benefit would be marginal at best. Future studies should therefore prioritize investigating C‐CTM versus MRI in deep‐pain‐negative dogs, where the potential for a clinically meaningful difference in outcomes is greatest.

MRI has been purported to enable precise preoperative patient selection, planning of decompressive surgery, and prognostication.^3^, ^19^ In this study, we found no evidence that these theoretical advantages translated into differences in clinical decision‐making, as reflected by similar rates of conservative management, surgical approach, extent of decompression, and use of adjunctive procedures between groups. A previous study showed that the use of MRI led to a greater theoretical extent of decompression compared to non‐contrast CT using within‐dog comparisons.^8^ Our study design precluded direct within‐dog comparisons but we found no differences in surgical treatments between groups, including the extent of actual decompression and the use of adjunctive procedures. More importantly, we extend previous findings^8^ by demonstrating that both MRI and C‐CTM led to similar extents of decompression and use of adjunctive procedures, and to similar rates of recovery of ambulation with comparable safety profiles, bridging the gap between diagnostic imaging findings and clinical outcomes.

Beyond demonstrating clinical non‐inferiority, time‐adjusted economic analysis showed that the C‐CTM pathway resulted in cost savings, driven by the lower costs of the imaging procedure itself. This finding is consistent with prior economic observations.^7^, ^23^ The higher cost of MRI relative to non‐contrast CT with or without CT‐myelography is well‐established and reflects the fundamental technical and operational differences between imaging modalities including scanner acquisition costs, maintenance requirements, staffing requirements, and scan duration.^3^ Importantly, no significant differences were observed in non‐imaging costs between groups. This isolates the economic impact of imaging modality choice to the imaging procedure itself and shows that the imaging modality did not lead to downstream cost differences in surgical or perioperative management. In human medicine, overuse of advanced diagnostic imaging is a recognized global problem and a major contributor to low‐value healthcare spending.^43^ In several clinical contexts in people, MRI has been shown to be overutilized without corresponding improvements in patient‐centered outcomes.^44^, ^45^, ^46^, ^47^ When considered alongside the clinical findings of this study, routine use of MRI in this population may represent a more expensive imaging strategy that results in similar clinical outcomes. Conversely, C‐CTM achieves comparable clinical outcomes at lower cost and may be considered an appropriate first‐line imaging modality for the perioperative management of non‐ambulatory dogs with acute thoracolumbar intervertebral disc extrusion.

The equivalent clinical outcomes observed between C‐CTM and MRI reflect the cumulative result of multiple decision points throughout the patient journey, rather than the studied imaging modalities alone, and should be interpreted with the single‐institutional study design in mind. Each milestone of the patient journey from initial referral consultation to diagnostic imaging, to decompressive surgery, to follow‐up, and the practice patterns of the surgeons in this study are reported. Results may not be generalizable to hospitals or surgeons where case management is markedly different. Similarly, safety events are a cumulative result of hospital case‐mix, case selection, preoperative decision‐making, surgical execution, and varying decision thresholds to perform postoperative imaging and revision decompressive surgery. It is therefore challenging to attribute postoperative neurologic deterioration, development of PMM, and composite postoperative complications to a single variable.^29^ As previously reported, there are important practice pattern differences between board‐certified neurologists and surgeons which evolve over time.^1^, ^21^ In this study, there was a strong institutional tendency toward surgical management of acute non‐ambulatory thoracolumbar IVDE. Imaging was used to confirm the diagnosis and guide surgical planning, although the threshold for proceeding to surgery was relatively consistent across cases within the confines of the retrospective study design. However, decisions regarding adjunctive procedures such as fenestration and durotomy are often influenced by surgeon preference and institutional practice patterns, which may limit the extent to which imaging‐derived information directly determines their use. In Europe, non‐boarded clinicians practicing neurosurgery are under‐represented in the literature^21^ but were over‐represented in this study. The results of this study are not definitive proof that MRI provides no benefit in this patient population, and we suggest that further studies are required to define whether and how MRI may benefit non‐ambulatory chondrodystrophic dogs with acute thoracolumbar IVDE.

The findings of the present study are not necessarily discordant with the body of literature suggesting that MRI may be prognostic in dogs with acute severe spinal cord injury.^3^ In spinal neurology, MRI is considered the anatomic gold standard as it provides superior detail of spinal cord parenchyma and related structures,^6^, ^7^ but this quality alone is insufficient to recommend its routine use in acute thoracolumbar IVDE.^48^ Numerous studies report associations between specific MRI biomarkers, neurologic grade, and postoperative outcomes.^11^, ^12^, ^15^, ^49^, ^50^, ^51^, ^52^ However, by design, these studies cannot answer whether routine use of MRI over another imaging modality improves aggregate patient outcomes. A test's ability to see more does not inherently equate to enabling better care.^48^ This study challenges the assumption that MRI is necessary in this patient population, as far as clinical outcomes are concerned.^3^ A limitation of retrospective prognostic biomarker research is the potential for a self‐fulfilling prophecy, particularly in dogs with severe neurologic injury. Clinical decision‐making, including euthanasia at presentation or following imaging based on perceived prognosis, may preclude observation of the true clinical course. This can result in selective exclusion of the most severely affected cases from outcome analyses, potentially biasing reported associations between imaging biomarkers and clinical outcomes. This phenomenon has been described in other veterinary conditions and may contribute to limited generalizability of retrospective imaging biomarker studies.^53^, ^54^ This study provides a framework to address this with a study design that mitigates confounding by indication and the bias arising from selective euthanasia. Within this framework, where surgeons practiced minimal preoperative selection, we found no evidence that the routine use of MRI improved outcomes. However, this study cannot rule out a potential prognostic benefit with MRI within a highly selective patient triage strategy; what the optimal patient triage strategy is, has not been defined. While we cannot prove this from our study, we believe that the benefits of MRI are likely to accrue to only a relatively small subset of these dogs, particularly deep‐pain‐negative dogs, as suggested by Parry and others,^7^ or those dogs where the pretest probability of IVDE is not high.^3^

The practice patterns observed in this study align with recently reported clinical trends,^21^ supporting the generalizability of our findings to clinicians with similar approaches. Our sample population was representative of dogs predisposed to thoracolumbar IVDE, and the recovery rates we observed are consistent with previously reported benchmarks.^42^ The rate of adjunctive CT‐myelography and postoperative loss of deep pain sensation in our cohort was also similar to that in recent reports.^26^, ^29^ The study's most significant limitation is the inherent group imbalance from the natural experiment, where the quasi‐randomizing event was infrequent. Statistical power and precision of results are limited by the smaller C‐CTM group and the small number of deep‐pain‐negative dogs within this group. Acquisition of MRI studies was non‐standardized such that qualitative review of imaging findings and their association with decision‐making or clinical outcomes was not feasible. Relatedly, the clinical reasoning behind specific decisions was often not explicitly recorded, meaning our analysis could only examine the aggregate effects of decision‐making rather than specific clinical decisions. The modified Frankel score is inherently subjective and has less than perfect interobserver agreement.^55^ Some dogs did not receive follow‐up neurologic examination and were followed up remotely. The study was powered to detect non‐inferiority with a margin of −12%; however, an acceptable margin of non‐inferiority has not been defined. The cost data in this study only reflected the studied hospital's pricing and may not be applicable to other hospitals where the cost disparity between CT with or without myelography and MRI may be different. This study only evaluated a population of dogs with a high pretest probability of acute thoracolumbar IVDE, and does not apply to clinical presentations outside this scope, where MRI may be superior due to different diagnostic sensitivities for non‐IVDE myelopathies. Lastly, follow‐up CT‐myelography is required for cases with non‐diagnostic or equivocal non‐contrast CT findings, which requires facilities and expertise that may not be universally available.

Future studies should aim to replicate our findings, ideally through a randomized trial utilizing within‐dog comparisons of C‐CTM and MRI. If randomization is not possible, a multicenter quasi‐randomized natural experiment similar to ours is warranted. Further studies should report pre‐imaging and preoperative case selection to allow interpretation of any clinician selection biases. Where feasible, prospective studies should incorporate structured decision‐making frameworks and standardized image acquisition to isolate which specific decisions (the optimal decompressive approach, the targeted use of adjunctive procedures,^27^ or improved patient selection) are genuinely enhanced by the additional information MRI provides. Future comparative studies should continue to analyze outcomes using ITT and PP principles. The ITT analysis would show any aggregate benefit of an MRI pathway, including that from improved preoperative patient selection. The PP analysis, by examining only protocol‐adherent dogs that proceed to surgery, would better isolate the effect of imaging on the quality of surgical decision‐making and execution. Future studies examining the reasons for the shifts in practice patterns may also be useful to determine the factors influencing clinician decisions regarding choice of diagnostic imaging modality.

In conclusion, this quasi‐randomized natural experiment showed that C‐CTM is non‐inferior to MRI for achieving recovery of ambulation in non‐ambulatory chondrodystrophic dogs with acute thoracolumbar IVDE, with a similar safety profile at significantly lower cost. Given its accessibility relative to MRI, C‐CTM may be preferred as a first‐line imaging strategy in dogs with a high pretest probability of acute thoracolumbar IVDE. The routine use of MRI in this patient population may not be justified.

## AUTHOR CONTRIBUTIONS

Low D, BVetMed, CertAVP(GSAS), PGCertVPS, MRCVS: Study conception, study design, data collection, data analysis, interpretation of results, and manuscript writing. Markovic A, DVM, CertAVP, PGCertVPS, MRCVS: Data collection, data analysis, interpretation of results, manuscript writing. All authors approved the final manuscript.

## FUNDING INFORMATION

We wish to thank IVC Evidensia Research Fund, for providing open access funding for this study. The funder did not have any involvement in the scientific conduct of this study.

## CONFLICT OF INTEREST STATEMENT

No conflicts of interest to declare.

## Supporting information


**Data S1.** TREND Statement Checklist.

## Data Availability

The data that support the findings of this study are available from the corresponding author upon reasonable request.
